# The Crosskey-Davies Experiment and Onchocerciasis Control in West Africa

**DOI:** 10.1371/journal.pntd.0003223

**Published:** 2014-10-09

**Authors:** Jesse B. Bump

**Affiliations:** Department of International Health, Georgetown University, Washington, D.C., United States of America; Centers for Disease Control and Prevention, United States of America

Professionals and practitioners in global health often confront problems of planetary dimensions with comparatively meager resources. This imbalance is rarely more daunting than for those concerned with neglected tropical diseases (NTDs). By definition, NTDs do not receive the political and financial resources dedicated to the highest priorities in global health. Yet the death and disability attributed to them is no less meaningful for their victims, and the imperative to act is no less urgent for those willing to pick up the gauntlet.

Although it never attracted the resources or attention of malaria or HIV, onchocerciasis in Africa is rare among NTDs because it has been the target of two World Health Organization (WHO) programs and is now controlled in many countries, despite numerous obstacles such as a parasite that lives for 10–14 years and a vector capable of migrating hundreds of kilometers. Both control programs were underpinned by knowledge gained by many individuals working in relative isolation at different times and places around the sub-Sahara, typically with a primary focus on some other problem. This paper examines one such example, a rural Nigerian project from the 1950s, to demonstrate the public health progress that can be made even when personnel are limited and resources virtually nonexistent. In a story of happenstance, innovation, dedication, and careful analysis, it shows the difference the right investigator can make. It shows how three people discovered features of the epidemiology, transmission, and control of onchocerciasis in Africa that were among the many essential intellectual and practical contributions to the design of the Onchocerciasis Control Programme (OCP), which ran from 1974–2002 and largely defeated the disease in West Africa.

By the end of the 1940s, onchocerciasis was known to colonial investigators as a locally significant cause of blindness in some rural areas of Africa, but its study remained a matter of individual initiative. A quarter century later, it had become an internationally recognized problem targeted across most of West Africa by the foremost global authority in public health. Perhaps more than any other single effort, the Crosskey-Davies Experiment of 1954–1960 contributed to the scientific and technical basis of this transformation by developing disease transmission monitoring techniques that would serve as standards for the next half century and by demonstrating that local control based on larviciding was unlikely to succeed because as the density of flies fell, the infectivity of those surviving rose.

## Onchocerciasis before the Crosskey-Davies Experiment

In the first half of the 20th century, sleeping sickness dominated the rural health agenda in Belgian, British, and French areas. Of the three colonizers, the British had by far the most entomological capacity because their primary approach to sleeping sickness was to target its tsetse fly vector, whereas the French attacked the parasite itself with drugs and the Belgians tried to limit transmission through both drugs and by restricting the movement of potentially infected people. However, the British had almost never assigned entomologists to work on onchocerciasis.

Basic features of the transmission cycle of onchocerciasis were elucidated as a matter of personal curiosity by sleeping sickness entomologists, as in 1925 when Donald Blacklock identified the vector fly as *Simulium damnosum*. Physicians and ophthalmologists assigned to sleeping sickness were responsible for much of what was known about the distribution and symptoms of onchocerciasis because the two diseases were sometimes coendemic. Surveys connected to troop conscription or sleeping sickness had discovered pockets of blindness, and although the disease was never a priority, isolated investigators had begun to suspect it might be a major public health problem, including Jean Hissette in the Belgian Congo in 1932 [1], Pierre Richet in Upper Volta in 1939 [2], and Harold Ridley and B. B. Waddy in the Gold Coast in the 1940s [3], [4].

Emerging knowledge of the disease's distribution and consequences inspired many efforts to control it [5]–[8]. Marcel Wanson notched an early victory by eliminating *S. damnosum* in Leopoldville (now Kinshasa) with an aerial fogging campaign [9]. Although the flies eventually returned, the disease had died out. However, most attempts elsewhere showed more temporary effects. Elimination of flies at Jinja, Uganda, initially seemed possible, but over the years the area was repeatedly reinfested [7]. Results were mixed even with intensive, combined approaches. One project in Chad included four points of attack: killing adult flies with insecticide fogging, killing larval flies by dosing rivers, removing adult worm nodules surgically, and treating patients with two drugs [10, unpublished data]. Nevertheless, the flies kept returning—later investigators speculated their source as another site more than 100 miles distant [11].

## Crosskey Arrives: Discovering the Problem's Scale

Roger W. Crosskey entered the Sleeping Sickness Service in the British colonial administration of Nigeria in 1951, having taken a bachelor's degree in zoology and applied entomology the year before at the Imperial College of Science and Technology. At the time, *Simulium* blackflies were little known to mainstream entomologists, and the genus was not covered prominently in entomological or parasitological coursework. As elsewhere, onchocerciasis and *Simulium* research in Nigeria had their roots in sleeping sickness work, but here such efforts heavily emphasized bush clearing because some believed that the tsetse would not cross large barren areas [12].

Crosskey's early duties included supervising a bush clearing team of 500 men, a boring task with no entomology involved. During a lapse in attention to the laborers, Crosskey noticed *S. damnosum* and later applied for permission to study it. His survey of blackflies in the Galma valley covered about 1,000 square miles and included catching 13,000 flies. Of these, he dissected 1,200, finding that up to 20% of flies carried the *Onchocerca* parasite during the rainy season [13].

Around the same time, the Nigerian Colonial Medical Service received its first ophthalmologist, Frank Budden. Budden found blindness caused by onchocerciasis during his rounds to rural dispensaries and subsequently began to survey the problem [14]. His results coupled with Crosskey's findings led to comprehensive investigations by both men in the early 1950s.

Crosskey and Budden communicated regularly, and each would follow up where the other found flies or blindness. Budden visited every province in Northern Nigeria, examined thousands of patients, and discovered infection rates of up to 95% and reduced vision or blindness of over 40% among older men in some communities. Budden's was not the first large onchocerciasis survey in Africa, but he broke new ground by calculating standardized rates of infection and blindness and using 1952 census data to extrapolate his findings for all of Northern Nigeria. This was the first assessment of onchocerciasis as a national public health problem. Nearly 20,000 people were blind, he calculated, and 339,000 were suffering other effects of infection, including skin disease and diminished sight [15].

In parallel, Crosskey covered all the likely bionomic zones—about three quarters of Northern Nigeria's 281,000 square miles [16]. As of 1952, he collaborated with his wife, Peggy Crosskey, also a trained entomologist. In the rains of 1953 alone (June through October), the Crosskeys collected 1,680 flies, finding an average 19.7% with developing or fully infective *Onchocerca volvulus* larvae. In some weeks, more than 30% had been infective, and in every catch, at least 10% were infective. The Crosskeys also found that infective flies caught away from breeding sites were just as plentiful as those close by [17].

With endemicity well established by both entomology and ophthalmology, disease control remained as the next important task, and “towards the end of 1954, the Medical Department, as a result of [the Budden and Crosskey] surveys, became interested in the possibility of establishing a pilot project for the control of *S. damnosum* to assess the prospects for the control of this insect… ” [18]. At the same time, Budden had also been collaborating with others in the British Cameroons to study the symptoms of onchocerciasis in relation to the distribution of parasites within the body. That work eventually became another factor helping to focus international attention on onchocerciasis in West Africa by showing that the blinding form of the disease was concentrated there [19], [20].

## The Crosskey-Davies Control Experiment 1954–1960

The Crosskey-Davies experiment included dosing breeding grounds with dichlorodiphenyltrichloroethane (DDT) to reduce the blackfly population and epidemiological surveillance by skin snipping and fly catching. It was different from previous attempts to control *S. damnosum* because it monitored the effects of controlling fly breeding on both the fly population and disease in the human population. Others had tried to eliminate *S. damnosum* in isolated foci, but this was a test of control in the middle of many overlapping foci, a setting typical in West Africa. Also, no one had attempted to evaluate the effect of control on disease in the human population. In settings in which eliminating the vector was not feasible, this was an important question [18].

The experiment was run in Abuja, at the time a small country town but now a busy area renamed Suleja west of the Nigerian capital that took its name in the 1970s. A strong working relationship with the Emir was crucial to the effort. A culturally sensitive arrangement was made whereby Roger would snip the skin of men and Peggy would take samples from the women. As the Crosskeys made their rounds, the Emir accompanied them with the tax rolls, lending a certain authority to the undertaking and enforcing full attendance. This feature of the experience is especially notable for its contrast with the problems encountered more recently in polio programs in Nigeria.

Two innovations came from the Crosskey-Davies experiment that changed the future of onchocerciasis control: a transmission monitoring method and a finding suggesting that local-scale control programs would not succeed.

Monitoring the impact of vector control on onchocerciasis transmission in the human population is not practicable at the onset of vector control for two reasons. First, new infections take up to 30 months to develop, making annual incidence measurement a poor option. Second, the adult parasite lives in the human host for a decade or more, meaning that annual prevalence changes are also barely measurable. This therefore means that measuring disease incidence is an unreliable indicator in the early stages of vector control.

Measuring the transmission of onchocerciasis had never been done before, but the Crosskeys developed an approach based on a hallmark of British sleeping sickness control, the so-called “fly-round” [21]. The adapted method involved defined fly catching points that were visited regularly, as opposed to the original version, which employed movements over standard distances because the tsetse is attracted by motion. On each visit, fly bait would be deployed for a roughly standard time. The flies caught would reveal data about the fly population. Dissecting the flies would reveal transmission potential [22].

The Crosskey adaptation of the fly-round formed the template for measuring onchocerciasis transmission and was later used by OCP throughout its three decades. As of the 1955 season, 43 catching points had been established. These were typically visited by two or three men—up to five in later years—who would expose their legs for 15 minutes and catch flies thereby attracted. The number of flies caught and the number of *Onchocerca* larvae they contained could be compared over time to measure changes in transmission potential from year to year and over the five years of the project [22].

The rest of the answer to the original proposition—could onchocerciasis be controlled in areas subject to blackfly reinfestation—depended on measuring changes in the disease burden. That could be determined accurately by skin snips, and comparing standardized snips taken over time was a way to measure changes [19], [23]. The Crosskeys took thousands of standardized skin snips over the years; for instance in 1957, the Crosskeys snipped 11,000 people in 82 villages inside and outside the control zone. Others contributed as well. In 1960, the rural health superintendent returned to snip 12,234 in 70 villages to gather post-control data [24].

Finding answers in the voluminous data rested on a painstaking analysis by John B. Davies, another former sleeping sickness entomologist who took over the project in 1959. Davies began by hand assembling a comparable dataset from a subset of 37 villages common to all snipping rounds—no small task because village names were spelled phonetically, were sometimes changed, and sometimes villages moved; “for example, Laiba, on the river Tapa, lay on the northern bank in 1957, but during 1959 the entire village of some 130 persons moved about two miles across the river to settle on the southern side” [24].

As expected, DDT larviciding brought declines in the number of flies captured, but the analysis revealed two large surprises. For boys, the mean earliest infection was not affected at all, and for girls, the earliest mean infection occurred at 5.7 years of age, a year earlier than before control. One reason was that although there were far fewer flies, the proportion of those carrying the parasite rose sharply, probably because the captured flies were older on average and had had more chances to ingest the parasite, Davies believed [24], [25]. Using pre- and post-control data, Davies calculated the number of infective bites per day, factoring in both reductions in fly density and increases in fly infectivity. Though the fly population plummeted by about 90%, increased infectivity meant that the number of infective bites per day declined by only half, still easily sustaining transmission (Table 1).

**Table 1:** Infective bites per day before and after control [25].

Period	Mean fly density per boy-hour (FBH)	Estimated bites per day (FBH * 10 hours)	Infection rate (%)	Estimated number of infective bites per day
July and August 1955 (pre-control)	12.6	126	0.7	0.88
July and August 1961 (post-control)	1.37	13.7	3.14	0.47

## Conclusions

The Crosskey-Davies control project set the standard for larviciding programs to come and shows how a few people with minimal resources can advance the fight against NTDs. The adapted blackfly round, standardized skin snipping, and meticulous record keeping and analysis were all important elements in OCP's strategy. By testing the possibility of control in an area subject to reinvasion and demonstrating how the surviving flies' infectivity rose, the project showed that local fly control efforts were unlikely to offer a sufficient long-term public health solution.

Local surveys by many investigators had shown the disease to be regionally prevalent, but the Crosskey-Davies project was an important demonstration that overlapping transmission zones would have to be attacked simultaneously. The regional dimensions of transmission in West Africa had special consequences because it required supranational authority to manage an effective control program. On the strength of research by many investigators—including those profiled here—and the advocacy of Pierre Richet, B. B. Waddy, and others, the World Health Organization eventually answered this call.
